# The effect of psychological richness on the meaning in life of college students: the chain mediating effect of sense of coherence and self-compassion

**DOI:** 10.3389/fpsyg.2025.1664328

**Published:** 2025-10-09

**Authors:** Qingsen He, Yixin Chen, Shan Cao

**Affiliations:** ^1^School of Nursing, Henan University of Chinese Medicine, Zhengzhou, China; ^2^School of Medicine, Henan University of Chinese Medicine, Zhengzhou, China

**Keywords:** psychological richness, sense of coherence, self-compassion, meaning in life, college students

## Abstract

**Background:**

Psychological richness is an important factor affecting the meaning in life of college students. Although previous studies have confirmed the association between psychological richness and meaning in life, its mechanism remains unclear.

**Objective:**

This study aimed to examine the relationship between psychological richness and the meaning in life of college students, and the chain mediating role of the sense of coherence and self-compassion in such a relationship.

**Methods:**

This study surveyed 2,671 college students in Henan Province, China, using the Psychologically Rich Life Questionnaire, Sense of Coherence Scale, Self-Compassion Scale-Short Form, and Meaning in Life Questionnaire.

**Results:**

(1) Psychological richness was significantly correlated with sense of coherence (*r* = 0.385, *p* < 0.001), self-compassion (*r* = 0.439, *p* < 0.001), and meaning in life (*r* = 0.296, *p* < 0.001); sense of coherence was correlated with self-compassion (*r* = 0.478, *p* < 0.001) and meaning in life (*r* = 0.375, *p* < 0.001); self-compassion was correlated with the meaning in life (*r* = 0.402, *p* < 0.001); (2) The mediating effect analysis showed that the mediating effect of sense of coherence (95%CI: 0.060 ~ 0.104), self-compassion (95%CI: 0.056 ~ 0.100) and the chain mediating effect of sense of coherence and self-compassion (95%CI: 0.026 ~ 0.047) were significant.

**Conclusion:**

Psychological richness can not only directly predict college students’ meaning in life, but also indirectly predict their meaning in life through the chain mediating effect of sense of coherence and self-compassion. This research provides valuable suggestions for universities, which will help them adjust their mental health education policies and provide a solid scientific basis for enhancing the sense of meaning in life and improving the mental health status of college students.

## 1 Introduction

Meaning in life refers to an individual’s perception of their life goals and existence values, comprising two dimensions: the presence of meaning and the search for meaning (Steger et al., 2006). The presence of meaning is an individual’s subjective feeling of the degree to which their life is important, purposeful and valuable, and is an individual’s understanding of the nature of life; The search for meaning is a dynamic and active effort made by individuals to clarify and enhance their understanding of the meaning, importance and purpose of their lives (Steger et al., 2008). Both the presence of meaning and the search for meaning have a positive impact on individual mental health and subjective well-being (Li et al., 2021). Studies have shown that individuals with a strong sense of meaning in life tend to show higher psychological resilience, stronger psychological adjustment ability, and more positive social adaptation posture (Hu and Wang, 2023; Liu et al., 2024). In addition, meaning in life can also negatively predict suicidal ideation, which is an important protective and promoting factor in the development of individual physical and mental health (Zhong et al., 2024).

However, the meaning in life of college students in the real situation is not optimistic. Some students show a lack of meaning in life due to multiple challenges such as academic pressure, employment uncertainty and complex interpersonal relationships (Zhang et al., 2024). The lack of meaning in life will lead to anxiety, depression and other negative emotions, and even suicidal thoughts (Haugan and Dezutter, 2021; Tsai et al., 2020). Therefore, studying the influencing factors of college students’ meaning in life is of great significance to prevent college students’ psychological problems and promote individual healthy development.

As a new form of well-being, psychological richness is typically characterized by novel, interesting, diverse, perspective-changing, complex, uncertain and challenging life experiences (Besser and Oishi, 2020; Oishi et al., 2020). Individuals with rich psychology will seek novel experiences to enrich their lives through movies, music, travel, sports and art, and these novel experiences tend to make individuals have higher life satisfaction and meaning in life (Oishi et al., 2019; Xing et al., 2025). Although previous studies have confirmed that there is an association between psychological richness and meaning in life, its mechanism is still unclear. Therefore, this study intends to explore the impact of psychological richness on College Students’ meaning in life and its internal mechanism, in order to provide new perspectives and strategies for improving the meaning in life of college students.

### 1.1 Psychological richness and meaning in life

According to the Theory of Well-Being, psychological richness is the third form of happiness that is different from happiness and meaning (Oishi et al., 2020). As a new form of happy life, psychological richness emphasizes the experience of individuals in life, which is full of novelty, uncertainty and complexity, and is an ideal and important part of people’s self-description concept of a good life (Xu, 2025). Empirical studies have shown that an individual’s psychological richness is closely linked to their curiosity, openness to new experiences, and willingness to challenge the status quo (Oishi and Westgate, 2022). Complex, novel, challenging and perspective changing experiences can help stimulate the experience of psychological richness. Individuals with high psychological richness are more willing to try new things, show richer imagination and higher openness, and tend to have higher wisdom and richer life experience (Wang et al., 2022b).

Oishi et al. (2019) believe that a psychologically rich life is composed of diverse and interesting experiences. By experiencing diverse and interesting experiences, individuals change their perspectives, their cognition changes accordingly, their emotions are fully awakened, prompting them to experience intense and rich emotions, thereby enhancing their perception of the meaning in life of their own lives (Xu, 2025). Existing research has found that psychological richness can effectively enhance individuals’ meaning in life (Oishi et al., 2021). Self-Determination Theory believes that individuals are born with three basic psychological needs: the need for autonomy, competence and relatedness (Deci and Ryan, 2000). Individuals with high psychological richness bring themselves rich psychological experience by virtue of their novel experience and diverse perspectives, which can fully meet their basic psychological needs (Wang et al., 2022a). When these needs are met, individuals will experience a strong sense of existence, self-worth and belonging, thus showing a higher meaning in life (Guardia et al., 2000). Based on this, the following hypothesis is proposed:

*H1:* Psychological richness positively predicts the meaning in life of college students.

### 1.2 The mediating role of sense of coherence

Sense of coherence is the core concept of the Salutogenic Model. It refers to a universal, dynamic and continuous confidence held by individuals in the face of internal and external environmental stimuli. It comprehensively reflects the overall feeling and cognition of individuals on life, and is a stable psychological tendency within individuals (Antonovsky, 1993). Sense of coherence includes three dimensions: comprehensibility, manageability, and meaningfulness. Comprehensibility refers to individuals’ perception that stimuli from internal and external environments are structured, predictable, and interpretable; manageability refers to the ability of individuals to obtain sufficient resources to cope with the demands brought about by these stimuli; meaningfulness refers to the fact that individuals believe that these stimuli and demands are a challenge and worth participating in and investing in (Eriksson, 2022). Brett et al. (2023) believes that sense of coherence can enable college students to fully mobilize psychosocial resources such as personality and social support to ensure that they can successfully cope with and adapt to adversity, which is the decisive factor to improve their happiness and promote their physical and mental health development.

As the key to the development of sense of coherence, general coping resources are resources that can be used to effectively cope with stress in individuals, groups and society (Hewis, 2023). Individuals gain healthy and positive life experience through the interaction between general coping resources and social environment, thereby enhancing the sense of coherence (Li et al., 2022). Individuals with a high level of psychological richness can actively carry out cognitive reconstruction and change perspective when facing difficulties, and are confident in their ability to cope with life challenges and internal and external stimuli (Oishi and Westgate, 2022). When facing stressful events in life, they tend to regard them as understandable and controllable (Liu et al., 2025). This positive cognitive style helps individuals find meaning from life events, thereby enhancing their sense of coherence.

Sense of coherence, as an internal psychological resource, can enable individuals to maintain internal stability and balance in the face of the uncertainty of stress events, help individuals relieve stress, stimulate them to take active action and find the goal and meaning of life (Bargehr et al., 2023). The buffer model of Olsson et al. (2002) indicates that sense of coherence plays a buffering role between negative life events and individuals’ physical and mental development, enabling individuals to be less affected or protected from external stress. Individuals with a high level of sense of coherence can mobilize more protective resources when facing negative events, reduce the negative impact brought by external pressure, help individuals maintain a positive psychological state, and thereby experience a higher meaning in life (Yao et al., 2023). Therefore, based on the above content, this study proposes the following hypotheses:

*H2:* Sense of coherence plays a mediating role between psychological richness and meaning in life of college students.

### 1.3 The mediating role of self-compassion

Self-compassion refers to an individual’s ability to face negative situations such as stress, setbacks, and failures with an accepting, open, and non critical attitude, and view them as a part of the universal human experience, and it is a positive self-awareness attitude that includes three dimensions: self kindness, the sense of common humanity, and mindfulness (Neff, 2003). Self-kindness refers to an individual’s ability to treat themselves with kindness and care when faced with pain or failure, rather than being overly critical of themselves; the sense of common humanity refers to the individual’s ability to recognize that everyone makes mistakes, experiences pain, and sees mistakes and failures as part of the universal human experience; mindfulness refers to an individual’s ability to objectively view the difficulties and situations they face, and to recognize and accept their thoughts and feelings in a balanced manner, rather than avoiding and resenting them (Neff, 2023). Individuals with a high level of self-compassion ability can cope with stressful events more easily, reduce the interference of negative emotions, and show a positive attitude and higher life satisfaction (Mey et al., 2023).

Oishi and Westgate (2022) pointed out that psychological richness can promote positive emotions. When confronted with negative situations in life, individuals with a high level of psychological richness tend to maintain an optimistic attitude and adopt positive coping methods rather than criticism, resentment and evasion, which helps to enhance their level of self-compassion. In addition, a high level of psychological richness can also provide individuals with rich emotional and cognitive experience, help individuals see themselves and negative events in life more comprehensively and deeply, help them better understand and accept themselves, and thus improve the level of individual self-compassion(Li et al., 2023).

Self-compassion is a psychological resource to cope with daily troubles and life challenges (Dvořáková et al., 2019). When facing difficulties or pressures in life, individuals with a higher level of self-compassion can better show kindness, warmth and affirmation to themselves, thereby restoring emotional balance (Ewert et al., 2021). Oishi et al. (2021) demonstrated that self-compassion can enable college students to confront and overcome adversity, recover from it, and thereby generate a stronger sense of meaning in life. According to the Logotherapy Theory of Frankl (1985), the attitude adopted by individuals in adversity is one of the ways to obtain the meaning in life (Frankl, 1985). Individuals with a high degree of self-compassion can face negative events with a positive attitude, thereby having more opportunities to gain a sense of meaning in their negative experiences (Zipagan and Galvez Tan, 2023; Neff et al., 2007). Suh and Chong (2022) also found that self-compassion is a powerful predictor of an individual’s meaning in life. Therefore, based on the above discussion, this study proposes the hypothesis:

*H3*: Self-compassion plays a mediating role between psychological richness and meaning in life of college students.

### 1.4 The chain mediating role of sense of coherence and self-compassion

Sense of coherence and self-compassion both belong to the category of positive psychology, and there is a certain correlation between them. Antonovsky (1987) pointed out in the Salutogenic Model that stressors cover negative, neutral and positive types, so the impact of stress on individuals is not completely negative, but may also be beneficial to individual health. Individuals with a high level of sense of coherence can perceive and view the internal and external stimuli in life in an organized, predictable and easy to understand way, and take a positive attitude and action to face pressure and difficulties, so as to stabilize their emotions, and improve their self-compassion level (Ghaffari and Salami Chaharborj, 2020; Zhang et al., 2023). The research of Bedoria and Madrigal (2022) has shown that when individuals demonstrate the ability to manage and understand the sources of their own stress, they can give themselves care and attention (Bedoria and Madrigal, 2022). That is to say, the higher the level of sense of coherence, the stronger the ability of self-compassion. Mowlaie et al. (2017) also found that there was a positive correlation between individual sense of coherence and self-compassion. Thus, the following hypothesis is proposed.

*H4*: Sense of coherence and self-compassion play a chain mediating role between psychological richness and meaning in life of college students.

**Figure 1:**
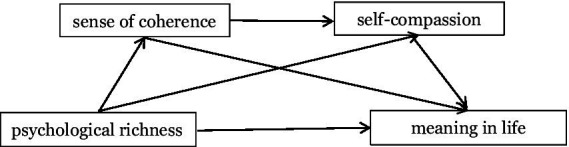
Hypothetical model.

In summary, this study will explore the relationship among psychological richness, sense of coherence, self-compassion and meaning in life of college students, and analyze the chain mediating role of sense of coherence and self-compassion between psychological richness and meaning in life of college students. The hypothetical model is shown in Figure 1.

## 2 Methods

### 2.1 Participants

A convenient sampling method was utilized to conduct a survey of Chinese college students in Henan Province using an integration of online questionnaires. When filling out the questionnaire, the researcher explained in detail the purpose, significance, intended use and filling guide of the survey to the participants to ensure the accuracy and validity of the data, and obtained the informed consent of the subjects before the test. The investigation follows the principle of voluntariness and anonymity throughout the whole process. A total of 2,932 questionnaires were retrieved in this study. After eliminating 261 invalid questionnaires, 2,671 valid questionnaires were finally obtained, with an effective recovery rate of 91.10%. Among them, there were 1,079 males (40.40%) and 1,592 females (59.60%). The average age was 20.37 years old (SD = 1.42).

### 2.2 Measures

#### 2.2.1 Psychologically Rich Life Questionnaire

The Psychologically Rich Life Questionnaire was developed by Oishi et al. (2019). Chinese scholar Wang et al. (2022b) revised the questionnaire and verified that the Chinese version of Psychologically Rich Life Questionnaire has good reliability and validity in Chinese college students. The scale is mainly used to measure the psychological richness level of college students, and contains 12 items. An example of a question included, “My mental life is rich.” Participants rated each item on a 7-point Likert scale, with values ranging from 1 = Strongly disagree to 7 = Strongly agree. Higher scores indicate a higher level of psychological richness. In this study, the Cronbach’s *α* coefficient was 0.975.

#### 2.2.2 Sense of coherence scale

The sense of coherence scale (SOC-13) was developed by Antonovsky (1993). Bao and Liu (2005) translated and revised the scale. It is mainly used to measure the level of individual sense of coherence. The scale includes three dimensions: comprehensibility (5 items), manageability (4 items) and meaningfulness (4 items), with a total of 13 items. An example of a question included, “Do you often feel that you do not care about what’s happening around you?” Participants rated each item on a 7-point Likert scale, with values ranging from 1 = never to 7 = often, and five items are scored in reverse. Higher scores indicate a higher level of sense of coherence. In this study, the Cronbach’s *α* coefficient was 0.868.

#### 2.2.3 Self-Compassion Scale-Short form

The Self-Compassion Scale-Short Form (SCS-SF) was developed by Raes et al. (2011). Chinese scholar Huang et al. (2023) verified that the Chinese version of this scale has good reliability and validity among Chinese college students. The scale is mainly used to measure the self-compassion of individuals. The scale comprises 12 items across six dimensions: Self-kindness (2 items), Self-judgment (2 items), Common Humanity (2 items), Isolation (2 items), Mindfulness (2 items), and Over-identification (2 items). An example of a question included, “When something painful happens, I tend to overreact to it and exaggerate its influence.” Participants rated each item on a 5-point Likert scale, with values ranging from 1 = almost never to 7 = almost always. A total of six items in the three dimensions of Self-judgment, Isolation and Over-identification are reverse scoring questions. Higher scores indicate a higher level of self-compassion. In this study, the Cronbach’s *α* coefficient was 0.891.

#### 2.2.4 Meaning in Life Questionnare

The Meaning in Life Questionnare (MLQ) was developed by Steger et al. (2006). Liu and Gan (2010) revised the Chinese version of the scale. It is mainly used to measure the level of meaning in life of an individual. The scale includes two dimensions: the Search for Meaning (4 items) and the Presence of Meaning (5 items), with a total of 9 items. An example of a question included, “I am looking for a purpose or mission in my life.” Participants rated each item on a 7-point Likert scale, with values ranging from 1 = never to 7 = always. Higher scores indicate a higher level of meaning in life. In this study, the Cronbach’s *α* coefficient was 0.947.

### 2.3 Data analysis

SPSS and PROCESS software were used for statistical analysis. Cronbach *α* coefficient was used to evaluate the reliability of the scale. Pearson correlation coefficient was calculated to explore the relationship between variables. The chain mediation model was established by using Model 6 in SPSS PROCESS compiled by Hayes to test the chain mediation relationship among psychological richness, sense of coherence, self-compassion and meaning in life. Bootstrap method was used to test the significance level of mediating effect, and repeated sampling was conducted for 5,000 times.

## 3 Results

### 3.1 Common method bias test

Harman single factor test was used to test the common method deviation. The results showed that the amount of variance explained by the first factor was 29.61%, which is less than the critical value of 40%. Therefore, common method bias was not a significant concern in this study.

### 3.2 Correlation analysis

This study conducted a correlation analysis of psychological richness, sense of coherence, self-compassion and meaning in life among college students. The results showed that psychological richness was significantly correlated with sense of coherence (*r* = 0.385, *p* < 0.001), self-compassion (*r* = 0.439, *p* < 0.001), and meaning in life (*r* = 0.296, *p* < 0.001); sense of coherence was correlated with self-compassion (*r* = 0.478, *p* < 0.001) and meaning in life (*r* = 0.375, *p* < 0.001); self-compassion was correlated with the meaning in life (*r* = 0.402, *p* < 0.001). The results are shown in Table 1.

**Table 1:** Means, standard deviations, and correlations among study variables.

Item	x̄ ± s	1	2	3	4
1. Psychological richness	53.668 ± 14.217	1			
2. Sense of coherence	58.491 ± 10.983	0.385***	1		
3. Self-compassion	38.741 ± 4.946	0.439***	0.478***	1	
4. Meaning in life	42.146 ± 10.613	0.296***	0.375***	0.402***	1

*** indicates *p* < 0.001.

### 3.3 Mediation effect test

Psychological richness was included as the independent variable, sense of coherence and self-compassion were included as mediating variables, and meaning in life was included as the dependent variable. The specific results of the regression analysis are presented in Table 2. Psychological richness positively predicted sense of coherence (*β* = 0.385, *p* < 0.001), self-compassion (*β* = 0.300, *p* < 0.001), and meaning in life (*β* = 0.102, *p* < 0.001). Sense of coherence positively predicted self-compassion (*β* = 0.363, *p* < 0.001) and meaning in life (*β* = 0.214, *p* < 0.001). Self-compassion positively predicted meaning in life (*β* = 0.256, *p* < 0.001).

**Table 2:** Regression analysis of variable relationships in the mediation model.

Regression equation		Overall fitting index			Significance of regression coefficient		
Result variable	Predictive variable	*R*	*R* ^2^	*β*	95% Confidence interval		*t*
					Lower	Upper	
Sense of coherence	Psychological richness	0.385	0.148	0.385	0.350	0.420	21.550***
Self-compassion	Psychological richness	0.553	0.305	0.300	0.265	0.334	17.137***
	Sense of coherence			0.363	0.329	0.397	20.766***
Meaning in life	Psychological richness	0.462	0.213	0.102	0.063	0.140	5.178***
	Sense of coherence			0.214	0.174	0.253	10.652***
	Self-compassion			0.256	0.215	0.296	12.403***

*** indicates *p* < 0.001.

**Figure 2:**
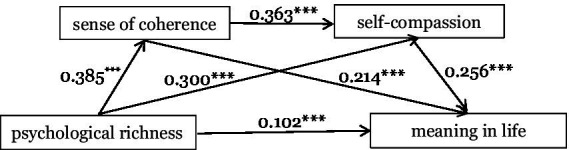
The chain mediation effect of sense of coherence and self-compassion.

The test of the chain mediating effect was conducted by repeated sampling 5,000 times using the non-parametric Bootstrap method. The results are shown in Table 3. The results show that the confidence interval for the direct effect of psychological richness on meaning in life does not include 0. It indicates that psychological richness has a significant direct effect on meaning in life, with a direct effect value of 0.102. Furthermore, the Bootstrap 95% confidence intervals of the mediating effect values of sense of coherence and self-compassion do not contain 0. It shows that sense of coherence and self-compassion are mediating variables between psychological richness and meaning in life. Specifically, the mediating effect of sense of coherence and self-compassion consists of three paths. Path 1: Psychological richness → Sense of coherence → Meaning in life; Path 2: Psychological richness → Self-compassion → Meaning in life; Path 3: Psychological richness → Sense of coherence → Self-compassion → Meaning in life. The mediating effect values of Path 1, Path 2 and Path 3 are 0.082, 0.076 and 0.036, respectively. In this study, the total indirect effect was 0.194, the total effect was 0.296, and the total indirect effect accounted for 65.541% of the total effect. The chain mediation model is shown in Figure 2.

**Table 3:** Mediating effect test of sense of coherence and self-compassion.

	Effect value	Bootstrap SE	95% confidence interval	
			Lower	Upper
Total effect	0.296	0.019	0.260	0.332
Indirect effect 1	0.082	0.012	0.060	0.104
Indirect effect 2	0.076	0.011	0.056	0.100
Indirect effect 3	0.036	0.006	0.026	0.047
Direct effect	0.102	0.020	0.036	0.140

## 4 Discussion

This study comprehensively examined the relationship between psychological richness, sense of coherence, self-compassion and meaning in life among college students. The results showed that psychological richness can affect college students’ meaning in life through sense of coherence and self-compassion, respectively. In addition, psychological richness can also affect college students’ meaning in life through the chain mediating effect of sense of coherence and self-compassion. This research provides a new perspective and scientific basis for understanding and enhancing the meaning in life of college students, and has important theoretical and practical value.

### 4.1 Relationship between psychological richness and meaning in life

This study found that psychological richness can significantly and positively predict the meaning in life of college students, supporting Hypothesis 1, which is consistent with previous research results (Oishi et al., 2021). The results also further support the view of the Self-Determination Theory. Individuals with psychological richness can independently explore and choose various life experiences, and obtain growth opportunities and positive and stable interpersonal relationships in these experiences, so as to fully meet their own needs for autonomy, competence and relevance. The full satisfaction of these three basic psychological needs can significantly improve the meaning in life of individuals (Zhang et al., 2022). Research has found that psychological richness can promote positive emotions (Oishi et al., 2019). According to the Broaden-and-Build Theory of positive emotions by Fredrickson (2001), the attention and cognitive expansion caused by positive emotional experience helps individuals cope with daily life events and adversity, and promotes them to find positive meaning (Fredrickson, 2001). That is to say, individuals with high levels of psychological richness have richer positive emotional experience, which can help individuals accumulate lasting personal and psychological resources, so as to help them better cope with life events and adversity, and perceive the positive meaning in life. In addition, the wisdom and interesting experience given to individuals by psychological richness can continuously expand their knowledge boundaries and make their thinking more flexible (Oishi and Westgate, 2022). Therefore, individuals with a high level of psychological richness can flexibly apply their knowledge to different situations of life, help them solve practical problems, and gradually improve their perception of their own value in solving problems, so as to enhance their meaning in life. In addition, as a new generation group, college students pay more attention to experiential life and like to pursue rich and interesting life experience. Zhang et al. (2016) pointed out that rich life experience is one of the main sources of college students’ meaning in life. The core of psychological richness is life experience, so individuals with high psychological richness can experience a higher level of meaning in life.

### 4.2 Mediating role of sense of coherence

This study also found that sense of coherence played a mediating role between psychological richness and college students’ meaning in life, supporting Hypothesis 2. Conservation of Resources Theory believes that individuals have the motivation to acquire, maintain, protect and cultivate personal resources to protect themselves and support social relations (Hobfoll, 1989). Individuals with psychological richness can accumulate a large number of useful resources for themselves, such as rich knowledge, a wide range of interests, and diverse emotional and cognitive experiences. These resources help individuals better understand and deal with various situations in life, enhance their control over life, and thereby improve the sense of coherence. Psychological richness can also increase individual wisdom and enhance individual adaptability (Oishi and Westgate, 2022; Wang et al., 2022a). In life, individuals with a higher level of psychological richness are more willing to take the initiative to seek opportunities for challenges and learning, and adjust their emotions and attitudes during challenges and learning. This adaptive ability helps to improve their confidence in coping with life challenges and pressures, and then enhance their sense of coherence.

Sense of coherence can affect individuals’ evaluation of their own resources and perception of the meaning of life in the face of internal and external environmental stimuli (Jasiński et al., 2025). Individuals with strong sense of coherence can clarify the nature of problems in cognition and emotion and are willing to face them. At the same time, they use their own and environmental resources to actively respond, and regard difficulties and setbacks as challenges and opportunities for growth, which is conducive to improving the individual’s perception of the meaning in life (Yao and Xue, 2021). According to the Stress and Stress Theory, when individuals face pressure or life events, they will first make a cognitive assessment and coping expectation on the stressors or life events, and then make a response (Zhang et al., 2021). Individuals with a high level of sense of coherence believe that stressors or life events are structured and predictable. They can actively mobilize their own resources to deal with them, reduce the negative impact of stress events on themselves, and clarify their own goals, find their own value, and ultimately enhance their meaning in life.

### 4.3 Mediating role of self-compassion

The results of this study showed that self-compassion played a mediating role between psychological richness and meaning in life of college students, which supported Hypothesis 3. According to the Model of Creativity, individuals with high levels of psychological richness have creative personality characteristics, can view and handle life events and pressures from different perspectives, and reduce their negative effects, which helps to improve individuals’ sense of self-confidence and self-esteem, and increase their ability to effectively deal with life challenges, so as to improve their self-comparison (González Moreno and Molero Jurado, 2023; Liu et al., 2025). In addition, psychological richness can also help individuals cope with tragedies and other adverse events in life, and this ability to cope with pain can help improve the level of individual self-comparison to a certain extent (Oishi and Westgate, 2022; Liu et al., 2025).

Self-compassion can help individuals adapt to the challenges in life and recover from them, and it helps them reinterpret painful experiences as meaningful ones that can promote personal growth (Ferreira et al., 2021). Therefore, individuals with a higher level of self-compassion can positively evaluate negative experiences in life, thereby prompting them to transform emotional pain into positive emotions and find meaning in life (Pérez-Aranda et al., 2021; Chan et al., 2022). Existing studies have shown that when facing challenges and threats in life, self-compassion can help individuals make psychological adjustments (Matos et al., 2022). Individuals with a high level of self-compassion can face setbacks and failures with positive attitudes such as kindness and understanding, thereby minimizing the impact of self-threatening events and enhancing their perception of meaning in life (O'Dea et al., 2022). Furthermore, during difficult times, individuals with a high level of self-compassion will engage in self-warmth and self-care, helping themselves overcome difficulties and regain vitality, thereby reducing their perception of pain and enhancing their perception of the meaning in life (Chan et al., 2022).

### 4.4 The chain mediating role of sense of coherence and self-compassion

This study also found that sense of coherence and self-comparison played a chain mediating role between psychological richness and college students’ meaning in life, which supported Hypothesis 4. Individuals with high psychological richness can understand themselves and the world around them from different levels, thereby better coping with internal and external environmental stimuli and showing a higher sense of coherence. Sense of coherence is one of the most critical determinants of individual success in coping with stress (Nosratabadi et al., 2023). Individuals with a strong sense of coherence can use more adaptive strategies to deal with stress, thereby enabling them to exhibit a good health-oriented attitude and actively participate in health-promoting behaviors, ultimately enhancing their self-compassion ability (Suraj and Singh, 2011). And self-compassion can improve the individual’s ability to recover and adapt to adversity, make them maintain calm emotions and feel a sense of security and belonging, so as to increase the individual’s sense of happiness and meaning in life (Nosratabadi et al., 2023).

## 5 Limitations and future research directions

This study has several limitations. First, this study adopts a cross-sectional research design, which cannot get the exact causal relationship. In the future, the effect of psychological richness on college students’ meaning in life and its internal mechanism can be further investigated by designing experiments to intervene or longitudinal tracking. Secondly, this study uses quantitative research methods, which makes it difficult to deeply understand the psychological experience and background factors of college students. Future research may consider incorporating qualitative research methods such as interviews, observations, and case studies to provide richer and more detailed explanations for the research. Finally, this study only examines the mediating role of sense of coherence and self-compassion between psychological richness and meaning in life among college students. Other potential variables that might affect this relationship are not included. Future studies may consider further exploring the role of other potential variables in the relationship between psychological richness and college students’ meaning in life, and exploring its influencing mechanism.

## 6 Conclusion

This study investigates the correlations between psychological richness, sense of coherence, self-compassion, and meaning in life. First, there are significant correlations between psychological Richness, sense of coherence, self-compassion, and meaning in life. Second, sense of coherence and self-compassion serves as a mediating factor between psychological richness and meaning in life. Third, sense of coherence and self-compassion function as chain mediators between psychological richness and meaning in life. The findings of this study substantially advance the theoretical understanding of the relationship between psychological richness and meaning in life. The research results will provide positive guidance for enhancing the sense of meaning in college students’ lives, reducing the incidence of psychological crises, improving their mental health status, and helping them develop healthily.

## Data Availability

The original contributions presented in the study are included in the article/supplementary material, further inquiries can be directed to the corresponding author.

## References

[ref1] Antonovsky A. (1987). Unraveling the mystery of health: how people manage stress and stay well: Jossey-Bass, 409–427 google schola, 2.

[ref2] Antonovsky A. (1993). The structure and properties of the sense of coherence scale. Soc. Sci. Med. 36, 725–733. doi: 10.1016/0277-9536(93)90033-z, PMID: 8480217

[ref3] Bao L. P. Liu J. S. (2005). The reliability and validity of Chinese version of SOC-13. Chin. J. Clin. Psychol. 13, 24–26. doi: 10.16128/j.cnki.1005-3611.2005.04.007

[ref4] Bargehr B. Fischer von Weikersthal L. Junghans C. Zomorodbakhsch B. Stoll C. Prott F. J.. (2023). Sense of coherence and its context with demographics, psychological aspects, lifestyle, complementary and alternative medicine and lay aetiology. J. Cancer Res. Clin. Oncol. 149, 8393–8402. doi: 10.1007/s00432-023-04760-9, PMID: 37079052 PMC10374667

[ref5] Bedoria C. J. S. Madrigal D. V. (2022). Sense of coherence, self-compassion, and mental well-being of senior high school students: an explanatory-sequential inquiry. Int. J. Multidiscip. Res. Growth Eval. 3, 239–253.

[ref6] Besser L. L. Oishi S. (2020). The psychologically rich life. Philos. Psychol. 33, 1053–1071. doi: 10.1080/09515089.2020.1778662

[ref7] Brett C. E. Mathieson M. L. Rowley A. M. (2023). Determinants of wellbeing in university students: the role of residential status, stress, loneliness, resilience, and sense of coherence. Curr. Psychol. 42, 19699–19708. doi: 10.1007/s12144-022-03125-8

[ref8] Chan K. K. S. Lee J. C. K. Yu E. K. W. Chan A. W. Leung A. N. M. Cheung R. Y.. (2022). The impact of compassion from others and self-compassion on psychological distress, flourishing, and meaning in life among university students. Mindfulness 13, 1490–1498. doi: 10.1007/s12671-022-01891-x, PMID: 35506030 PMC9050348

[ref10] Deci E. L. Ryan R. M. (2000). The “what” and “why” of goal pursuits: human needs and the self-determination of behavior. Psychol. Inq. 11, 227–268. doi: 10.1207/s15327965pli1104_01

[ref11] Dvořáková K. Greenberg M. T. Roeser R. W. (2019). On the role of mindfulness and compassion skills in students’ coping, well-being, and development across the transition to college: a conceptual analysis. Stress. Health 35, 146–156. doi: 10.1002/smi.2850, PMID: 30516320 PMC6491916

[ref12] Eriksson M. (2022). “The sense of coherence: the concept and its relationship to health” in The handbook of salutogenesis, 61–68. 36121981

[ref13] Ewert C. Vater A. Schröder-Abé M. (2021). Self-compassion and coping: a meta-analysis. Mindfulness 12, 1063–1077. doi: 10.1007/s12671-020-01563-8

[ref14] Ferreira C. Barreto M. Oliveira S. (2021). The link between major life events and quality of life: the role of compassionate abilities. Community Ment. Health J. 57, 219–227. doi: 10.1007/s10597-020-00638-z, PMID: 32440797

[ref9001] Frankl V. E. (1985). Man’s search for meaning. Simon and Schuster.

[ref15] Fredrickson B. L. (2001). The role of positive emotions in positive psychology: the broaden-and-build theory of positive emotions. Am. Psychol. 56, 218–226. doi: 10.1037/0003-066x.56.3.218, PMID: 11315248 PMC3122271

[ref16] Ghaffari M. Salami Chaharborj M. (2020). The relationships between sense of coherence and self-compassion to job stress with the mediating role of affective control. J. Res. Psychopathol. 1, 40–47. doi: 10.22098/jrp.2020.1031

[ref17] González Moreno A. Molero Jurado M. D. M. (2023). Creativity as a positive factor in the adolescence stage: relations with academic performance, stress and self-esteem. Behav. Sci. 13:997. doi: 10.3390/bs13120997, PMID: 38131853 PMC10740570

[ref18] Guardia J. G. L. Ryan R. M. Couchman C. E. Deci E. L. (2000). Within-person variation in security of attachment: a self-determination theory perspective on attachment, need fulfillment, and well-being. J. Pers. Soc. Psychol. 79, 367–384. doi: 10.1037/0022-3514.79.3.367, PMID: 10981840

[ref19] Haugan G. Dezutter J. (2021). “Meaning-in-life: a vital salutogenic resource for health” in Health promotion in health care–vital theories and research, 85–101. 36315724

[ref20] Hewis J. (2023). A salutogenic approach: changing the paradigm. J. Med. Imaging Radiat. Sci. 54, S17–S21. doi: 10.1016/j.jmir.2023.02.004, PMID: 36842893

[ref21] Hobfoll S. E. (1989). Conservation of resources: a new attempt at conceptualizing stress. Am. Psychol. 44, 513–524. doi: 10.1037/0003-066X.44.3.513, PMID: 2648906

[ref22] Hu J. Wang Y. (2023). Relationship between meaning in life and internalizing and externalizing problems of adolescents: the sequential mediating role of social connectedness and resilience. Chin. J. Health Psychol. 31, 1853–1859. doi: 10.13342/j.cnki.cjhp.2023.12.018

[ref23] Huang L. Y. Qu D. Y. Liang K. X. Ren Y. Z. Chi X. L. (2023). Longitudinal measurement invariance, validity, and reliability analysis of the self-compassion scale-short form among Chinese college students. Chin. J. Clin. Psychol. 31, 107–115. doi: 10.16128/j.cnki.1005-3611.2023.01.019

[ref24] Jasiński A. M. Derbis R. Rakoczy J. Wrzesińska M. (2025). The sense of coherence scale and relationships between sense of coherence, sociodemographic variables and chronic disease. Sci. Rep. 15, 18289–18214. doi: 10.1038/s41598-025-02998-6, PMID: 40419590 PMC12106626

[ref25] Li J. B. Dou K. Liang Y. (2021). The relationship between presence of meaning, search for meaning, and subjective well-being: a three-level meta-analysis based on the meaning in life questionnaire. J. Happiness Stud. 22, 467–489. doi: 10.1007/s10902-020-00230-y

[ref26] Li Y. X. Fan Q. J. Huang F. Y. Liu Y. N. (2023). Relationship between psychological richness and subjective well-being in college students: the chain mediating role of self-esteem and meaning in life. Adv. Psychol. 13:2726. doi: 10.12677/AP.2023.137336

[ref27] Li X. Y. Zhang J. Wu P. Sun L. (2022). Application of sense of coherence theory in maternity care. Health Res. 42, 662–666. doi: 10.19890/j.cnki.issn1674-6449.2022.06.013

[ref28] Liu S. S. Gan Y. Q. (2010). Reliability and validity of the Chinese version of the meaning in life questionnaire. Chin. Ment. Health J. 24, 478–482. doi: 10.3969/j.issn.1000-6729.2010.06.0212010

[ref29] Liu Y. Yang X. Liu Y. Wei C. Zhao J. Kong F. (2025). Bidirectional relationship between self-compassion and psychological richness: a two-wave longitudinal study. Appl. Psychol. Health Well Being 17:e12607. doi: 10.1111/aphw.12607, PMID: 39402984

[ref30] Liu Y. Zhou P. Y. Xiao M. L. Chen H. You Z. Q. Liu H. Y. (2024). Effects of family resilience on undergraduates' social adaption: a chain mediation analysis. Chin. J. Clin. Psychol. 32, 774–778. doi: 10.16128/j.cnki.1005-3611.2024.04.010

[ref31] Matos M. McEwan K. Kanovský M. Halamová J. Steindl S. R. Ferreira N.. (2022). Compassion protects mental health and social safeness during the COVID-19 pandemic across 21 countries. Mindfulness 13, 863–880. doi: 10.1007/s12671-021-01822-2, PMID: 35003380 PMC8724602

[ref32] Mey L. K. Wenzel M. Morello K. Rowland Z. Kubiak T. Tüscher O. (2023). Be kind to yourself: the implications of momentary self-compassion for affective dynamics and well-being in daily life. Mindfulness 14, 622–636. doi: 10.1007/s12671-022-02050-y, PMID: 36644400 PMC9823261

[ref33] Mowlaie M. Mikaeili N. Aghababaei N. Ghaffari M. Pouresmali A. (2017). The relationships of sense of coherence and self-compassion to worry: the mediating role of personal intelligence. Curr. Psychol. 36, 630–636. doi: 10.1007/s12144-016-9451-1

[ref34] Neff K. (2003). Self-compassion: an alternative conceptualization of a healthy attitude toward oneself. Self Identity 2, 85–101. doi: 10.1080/15298860309032

[ref35] Neff K. D. (2023). Self-compassion: theory, method, research, and intervention. Annu. Rev. Psychol. 74, 193–218. doi: 10.1146/annurev-psych-032420-031047 35961039

[ref36] Neff K. D. Kirkpatrick K. L. Rude S. S. (2007). Self-compassion and adaptive psychological functioning. J. Res. Pers. 41, 139–154. doi: 10.1016/j.jrp.2006.03.004

[ref37] Nosratabadi I. Ameri G. F. Iranmanesh S. Asadi N. (2023). Comparative study of self-compassion and sense of coherence in nurses of psychiatric hospitals. Front. Nurs. 10, 193–201. doi: 10.2478/fon-2023-0020

[ref38] O'Dea M. K. Igou E. R. van Tilburg W. A. Kinsella E. L. (2022). Self-compassion predicts less boredom: the role of meaning in life. Pers. Individ. Differ. 186:111360. doi: 10.1016/j.paid.2021.111360

[ref39] Oishi S. Choi H. Buttrick N. Heintzelman S. J. Kushlev K. Westgate E. C.. (2019). The psychologically rich life questionnaire. J. Res. Pers. 81, 257–270. doi: 10.1016/j.jrp.2019.06.010

[ref40] Oishi S. Choi H. Koo M. Galinha I. Ishii K. Komiya A.. (2020). Happiness, meaning, and psychological richness. Affect. Sci. 1, 107–115. doi: 10.1007/s42761-020-00011-z, PMID: 36042966 PMC9383031

[ref41] Oishi S. Choi H. Liu A. Kurtz J. (2021). Experiences associated with psychological richness. Eur. J. Personal. 35, 754–770. doi: 10.1177/0890207020962334

[ref42] Oishi S. Westgate E. C. (2022). A psychologically rich life: beyond happiness and meaning. Psychol. Rev. 129, 790–811. doi: 10.1037/rev0000317, PMID: 34383524

[ref43] Olsson U. Bergbom I. Bosaeus I. (2002). Patients’ experiences of the recovery period 3 months after gastrointestinal cancer surgery. Eur. J. Cancer Care 11, 51–60. doi: 10.1046/j.1365-2354.2002.00292.x, PMID: 11966835

[ref44] Pérez-Aranda A. García-Campayo J. Gude F. Luciano J. V. Feliu-Soler A. González-Quintela A.. (2021). Impact of mindfulness and self-compassion on anxiety and depression: the mediating role of resilience. Int. J. Clin. Health Psychol. 21:100229. doi: 10.1016/j.ijchp.2021.100229 33767736 PMC7957152

[ref45] Raes F. Pommier E. Neff K. D. Van Gucht D. (2011). Construction and factorial validation of a short form of the self-compassion scale. Clin. Psychol. Psychother. 18, 250–255. doi: 10.1002/cpp.702, PMID: 21584907

[ref46] Steger M. F. Frazier P. Oishi S. Kaler M. (2006). The meaning in life questionnaire: assessing the presence of and search for meaning in life. J. Couns. Psychol. 53, 80–93. doi: 10.1037/0022-0167.53.1.80

[ref47] Steger M. F. Kashdan T. B. Sullivan B. A. Lorentz D. (2008). Understanding the search for meaning in life: personality, cognitive style, and the dynamic between seeking and experiencing meaning. J. Pers. 76, 199–228. doi: 10.1111/j.1467-6494.2007.00484.x, PMID: 18331281

[ref48] Suh H. Chong S. S. (2022). What predicts meaning in life? The role of perfectionistic personality and self-compassion. J. Constr. Psychol. 35, 719–733. doi: 10.1080/10720537.2020.1865854

[ref49] Suraj S. Singh A. (2011). Study of sense of coherence health promoting behavior in north Indian students. Indian J. Med. Res. 134, 645–652. doi: 10.4103/0971-5916.90989, PMID: 22199103 PMC3249962

[ref50] Tsai F. J. Hu Y. J. Yeh G. L. Chen C. Y. Tseng C. C. Chen S. C. (2020). The effectiveness of a health promotion intervention on the meaning of life, positive beliefs, and well-being among undergraduate nursing students: one-group experimental study. Medicine 99:e19470. doi: 10.1097/MD.0000000000019470, PMID: 32150107 PMC7478399

[ref51] Wang Y. J. Ren X. P. Zhang L. (2022a). Psychological richness: a new form of well-being. Psychol. Tech. Appl. 10, 631–640. doi: 10.16842/j.cnki.issn2095-5588.2022.10.006

[ref52] Wang Y. J. Ren X. P. Zhang L. (2022b). Reliability and validity of the psychologically rich life questionnaire in college students. Chin. J. Clin. Psychol. 30, 936–939. doi: 10.16128/j.cnki.1005-3611.2022.04.035

[ref53] Xing L. L. Rao F. Zhong M. Wang J. (2025). Impact of college students psychological richness on career adaptability: chain mediation of growth mindset and career self-efficacy. Chin. J. Health Psychol. 33, 795–800. doi: 10.13342/j.cnki.cjhp.2025.05.030

[ref54] Xu X. Y. (2025). The impact of psychological richness on creativity among college students under the ecological psychological perspective and intervention strategies. Psychol. Mon. 20, 136–138. doi: 10.19738/j.cnki.psy.2025.07.037

[ref55] Yao M. T. Huang Z. C. Ma J. Y. (2023). Relationship among stress, sense of coherence, coping efficiency and life satisfaction of college students. Occup. Health 39, 671–680. doi: 10.13329/j.cnki.zyyjk.2023.0132

[ref56] Yao M. T. Xue Y. (2021). The influence of stress on the subjective well-being of college students: the mediating effect of sense of coherence. Occup. Health 37, 3247–3250. doi: 10.13329/j.cnki.zyyjk.20210922.003

[ref57] Zhang S. Feng R. Fu Y. N. Liu Q. He Y. Turel O.. (2022). The bidirectional relationship between basic psychological needs and meaning in life: a longitudinal study. Pers. Individ. Differ. 197:111784. doi: 10.1016/j.paid.2022.111784

[ref58] Zhang P. Peng L. Xu C. Zhang W. M. Liu Y. Li M. (2024). Effect of group psychological training based on gratitude and resilience on sense of meaning in life and psychological well-being among college students. J. Army Med. Univ. 46, 969–977. doi: 10.16016/j.2097-0927.202306041

[ref59] Zhang H. Sang Z. Chan D. K. S. Teng F. Liu M. Yu S.. (2016). Sources of meaning in life among Chinese university students. J. Happiness Stud. 17, 1473–1492. doi: 10.1007/s10902-015-9653-5

[ref9002] Zhang J. H. Shi M. Liu X. Y. (2021). Research on the correlation between life events, psychological consistency, and psyc hological harmony Modern communication.

[ref60] Zhang H. Y. Yang M. Q. Luo Y. F. (2023). Mediating effect of psychological consistency and positive coping style on emotional intelligence and mental health of undergraduate nursing interns in Sichuan Province. Occup. Health 39, 1529–1538. doi: 10.13329/j.cnki.zyyjk.2023.0295

[ref61] Zhong W. Liang Q. Yang A. Yan R. (2024). Why emotional neglect brings suicidal ideation? The mediating effect of meaning in life and the moderating effect of post-stress growth. Child Abuse Negl. 149:106700. doi: 10.1016/j.chiabu.2024.106700, PMID: 38382400

[ref62] Zipagan F. B. Galvez Tan L. J. T. (2023). From self-compassion to life satisfaction: examining the mediating effects of self-acceptance and meaning in life. Mindfulness 14, 2145–2154. doi: 10.1007/s12671-023-02183-8

